# From enrichment to interpretation: PS4-driven reclassification in Taiwanese inherited retinal degeneration

**DOI:** 10.1186/s40246-026-00923-0

**Published:** 2026-02-15

**Authors:** Yu-Shu Huang, Chien-Yu Lin, Yu-An Chen, Chieh-Yu Lee, Chang-Hao Yang, Jacob Shujui Hsu, Ta-Ching Chen, Pei-Lung Chen

**Affiliations:** 1 Graduate Institute of Clinical Medicine, College of Medicine, National Taiwan University, Taipei, Taiwan; 2 Department of Ophthalmology, National Taiwan University Hospital, Taipei, Taiwan; 3 Department of Computing and Data Sciences, Boston University, Boston, Massachusetts USA; 4 Department of Medical Research, National Taiwan University Hospital Hsin-Chu Branch, Hsinchu, Zhubei City, 302058 Taiwan; 5 Graduate Institute of Medical Genomics and Proteomics, College of Medicine, Medical College, National Taiwan University, Taipei, Taiwan; 6 Department of Ophthalmology, College of Medicine, National Taiwan University, Taipei, Taiwan; 7 Center of Frontier Medicine, National Taiwan University Hospital, Taipei, Taiwan; 8 Research Center for Developmental Biology and Regenerative Medicine, National Taiwan University, Taipei, Taiwan; 9 Department of Medical Research, National Taiwan University Hospital, Taipei, Taiwan; 10 Department of Medical Genetics, National Taiwan University Hospital, Taipei, Taiwan

**Keywords:** Inherited retinal degeneration, PS4 (case–control enrichment), ACMG/AMP classification, Ancestry-matched controls, Taiwan biobank, Variant interpretation

## Abstract

**Background:**

Inherited retinal degeneration (IRD) comprises a diverse group of monogenic disorders characterized by marked genetic and phenotypic heterogeneity. Although next-generation sequencing (NGS) enables the identification of candidate variants, many remain classified as variants of uncertain significance (VUS). Ancestry-matched population data can strengthen comparative evidence, and the emergence of national biobanks provides new opportunities to operationalize ACMG/AMP criterion PS4 through case–control analyses.

**Methods:**

We integrated an IRD cohort of 802 probands with whole-genome allele frequency data from 1,492 individuals in the Taiwan Biobank. An allele-based case–control framework was applied, assigning PS4 when the Haldane–Anscombe–corrected odds ratio was ≥ 5 and the 95% confidence interval excluded 1. Post-PS4 triage required variants to: (i) reside in IRD-associated genes, (ii) be rare in East Asian populations in gnomAD v4.1, and (iii) be annotated in RefSeq as exonic, untranslated regions, or splicing (± 20 bp). Baseline ACMG/AMP classifications were generated using GeneBe and finalized through expert curation.

**Results:**

Incorporation of PS4 substantially refined variant interpretation, upgrading two variants from Likely Pathogenic to Pathogenic and six from VUS to Likely Pathogenic. Homozygous exemplar variants, including *CNGB1* (NM_001297.5): c.2921T > G and *CFAP410* (NM_004928.3): c.340_351dup, demonstrated strong genotype–phenotype concordance with confirmatory sequencing, illustrating an end-to-end workflow from statistical enrichment to clinical reporting.

**Conclusion:**

An ancestry-aware case–control framework enables effective implementation of PS4 and improves the accuracy of IRD variant classification. This reproducible strategy supports the integration of population-specific genomic data into clinical workflows and is applicable to other monogenic disorders.

**Supplementary Information:**

The online version contains supplementary material available at 10.1186/s40246-026-00923-0.

## Introduction

Inherited retinal degeneration (IRD) comprises a group of monogenic disorders characterized by progressive loss of photoreceptors and retinal pigment epithelium, ultimately leading to visual impairment and blindness [1]. Pathogenic variants have been identified in more than 300 genes [2], giving rise to a wide clinical spectrum that includes rod–cone dystrophy, cone–rod dystrophy, macular dystrophy, Leber congenital amaurosis, and syndromic forms of retinal disease [3]. This diversity underscores the marked genotypic and phenotypic heterogeneity of IRD. In recent years, increasingly large IRD cohorts have been reported across diverse populations [4–6]. In parallel, many countries have established population biobanks that provide ancestry-resolved allele frequencies at a population scale [7–9].

The convergence of well-phenotyped disease cohorts and national biobanks offers new opportunities for ancestry-matched case–control analyses, in which patients serve as cases and biobank participants as population-matched controls. Within the ACMG/AMP variant interpretation framework [10], such analyses enable the application of the PS4 criterion, which evaluates variant enrichment in affected individuals relative to controls. In principle, ancestry-matched enrichment analyses can provide critical evidence to support reclassification of variants of uncertain significance (VUS), complementing segregation and functional data. However, despite its conceptual importance, PS4 remains underutilized in IRD variant interpretation, particularly in non-European populations. The lack of ancestry-matched control data and the absence of reproducible analytical workflows have limited the systematic implementation of PS4 in routine clinical genomics, leaving many potentially pathogenic variants unresolved.

Building on prior work from the Taiwan Inherited Retinal Degeneration Project (TIP), which characterized the genetic landscape and epidemiology of a national IRD cohort [11], the TIP dataset expanded to include 802 probands by June 2024. For ancestry-matched population frequencies, we leveraged high-coverage whole-genome sequencing data from the Taiwan Biobank, comprising 1,492 Taiwanese individuals [12]. In this study, we integrate a national IRD cohort with a population-matched biobank to systematically apply PS4 in a Taiwanese context. Our objectives are to quantify the incremental contribution of PS4 to ACMG/AMP variant classification and to establish a reproducible, ancestry-aware workflow that can be adapted to other monogenic disease programs with comparably sized cohorts.

## Materials and methods

### Patients

Between July 2015 and June 2024, we recruited 1,182 individuals from 937 families with suspected IRD. After comprehensive ophthalmic evaluation, including best-corrected visual acuity, electroretinography, color fundus photography, optical coherence tomography, and fundus autofluorescence imaging, 138 cases from 135 families were excluded. Exclusion criteria included non-IRD clinical diagnoses, atypical or unilateral phenotypes inconsistent with IRD, and incomplete clinical, genetic, or consent data that precluded reliable interpretation. Peripheral blood samples were collected from all participants after obtaining informed consent. Genomic DNA was extracted from peripheral blood mononuclear cells using the Gentra Puregene Blood Kit (QIAGEN, Hilden, Germany).

### TIP panel sequencing and data processing

All probands underwent targeted sequencing using an IRD gene panel developed by the Taiwan Inherited Retinal Degeneration Project (TIP), as previously described [11]. Four panel versions were used. Versions 1 to 3 targeted 233 monogenic IRD genes, while version 4 targeted 182 genes (Supplementary Table S1). Version 4 was streamlined to improve cost-effectiveness by removing genes that had not contributed to any confirmed diagnoses in earlier iterations. A detailed breakdown of panel versions, including gene content, capture completeness, and the number of samples sequenced with each version, is provided in Supplementary Table S2. Captured libraries were sequenced in paired-end mode on Illumina MiSeq or NextSeq 550 instruments (Illumina, San Diego, CA, USA). Raw FASTQ files were processed according to the Genome Analysis Toolkit (GATK) best practices using the Illumina DRAGEN Bio-IT Platform (v4.2). The workflow included alignment to the GRCh38 reference genome, duplicate marking, base-quality recalibration, and germline single nucleotide variant and indel calling. Per-sample genomic Variant Call Formats (gVCFs) were generated and jointly genotyped across the cohort using DRAGEN’s joint-calling workflow to produce a multi-sample Variant Call Format (VCF). Variant filtering was performed using DRAGEN’s built-in algorithms, and sequencing depth as well as coverage metrics were summarized. All variants were subsequently annotated with ANNOVAR [13].

### PS4 computation (case–control enrichment)

We assessed the ACMG/AMP criterion PS4 by evaluating whether each variant was enriched in cases compared with controls using an allele-based 2 × 2 contingency framework. For each variant, case-side allele counts (AC), allele numbers (AN), and allele frequencies (AF) were parsed from a VCF-derived key–value field. The reference-allele count in cases was defined as the total number of alleles observed in cases after subtracting the number of alternate alleles. Control allele counts and allele numbers were obtained directly from the Taiwan Biobank whole-genome sequencing dataset (TWB1492) [12] using variant-level, empirically observed AC and AN values. These control AC and AN values therefore reflect site-specific call rates and missingness rather than uniform or inferred denominators. Control reference-allele counts were calculated by subtracting the alternate-allele count from the total control allele number. For variants with an observed control alternate-allele count of zero, site-level callability was examined to confirm that the absence of alternate alleles reflected true absence in the control cohort rather than insufficient sequencing coverage. Odds ratios were computed using Haldane–Anscombe–corrected counts, and two-sided 95% confidence intervals as well as p-values were obtained using Fisher’s exact test. To account for multiple testing across all variants subjected to PS4 case–control analysis, q-values were calculated using the Benjamini–Hochberg false discovery rate (FDR) procedure. FDR-adjusted q-values were used as a sensitivity analysis to assess the robustness of PS4-enriched variants, but were not applied as mandatory thresholds for PS4 assignment, consistent with ACMG/AMP 2015 guidelines. For variants located on chromosome X, we additionally performed sex-stratified PS4 analysis. Male (XY) and female (XX) samples were analyzed separately, with allele counts, allele numbers, and odds ratios calculated independently for each sex to account for sex-specific ploidy and potential imbalance in cohort composition.

### Variant prioritization after PS4 enrichment

After identifying PS4-enriched variants, defined as those with an odds ratio ≥ 5 and a 95% confidence interval that excluded 1 [10], we applied the following prioritization steps. We first retained variants captured by the TIP IRD gene panel and removed those not mapped to genes included in the panel. Variants present at an allele frequency greater than 0.01 in East Asian populations in either the gnomAD genome or exome datasets (v4.1) were excluded. Based on RefSeq annotations, we retained variants classified as exonic, untranslated regions (UTR), or splicing (± 20 bp), and removed variants assigned to other categories. For all variants that passed these filters, pathogenicity assessment followed ACMG/AMP guidelines. We used the GeneBe platform (https://genebe.net/) to generate evidence tags and subsequently performed manual review for each variant to adjudicate the evidence, adding PS4 where applicable. Final ACMG classifications were determined after manual review and summarized in Supplementary Tables S3 and S4.

## Results

### PS4-driven enrichment and impact on classification

**Fig. 1:**
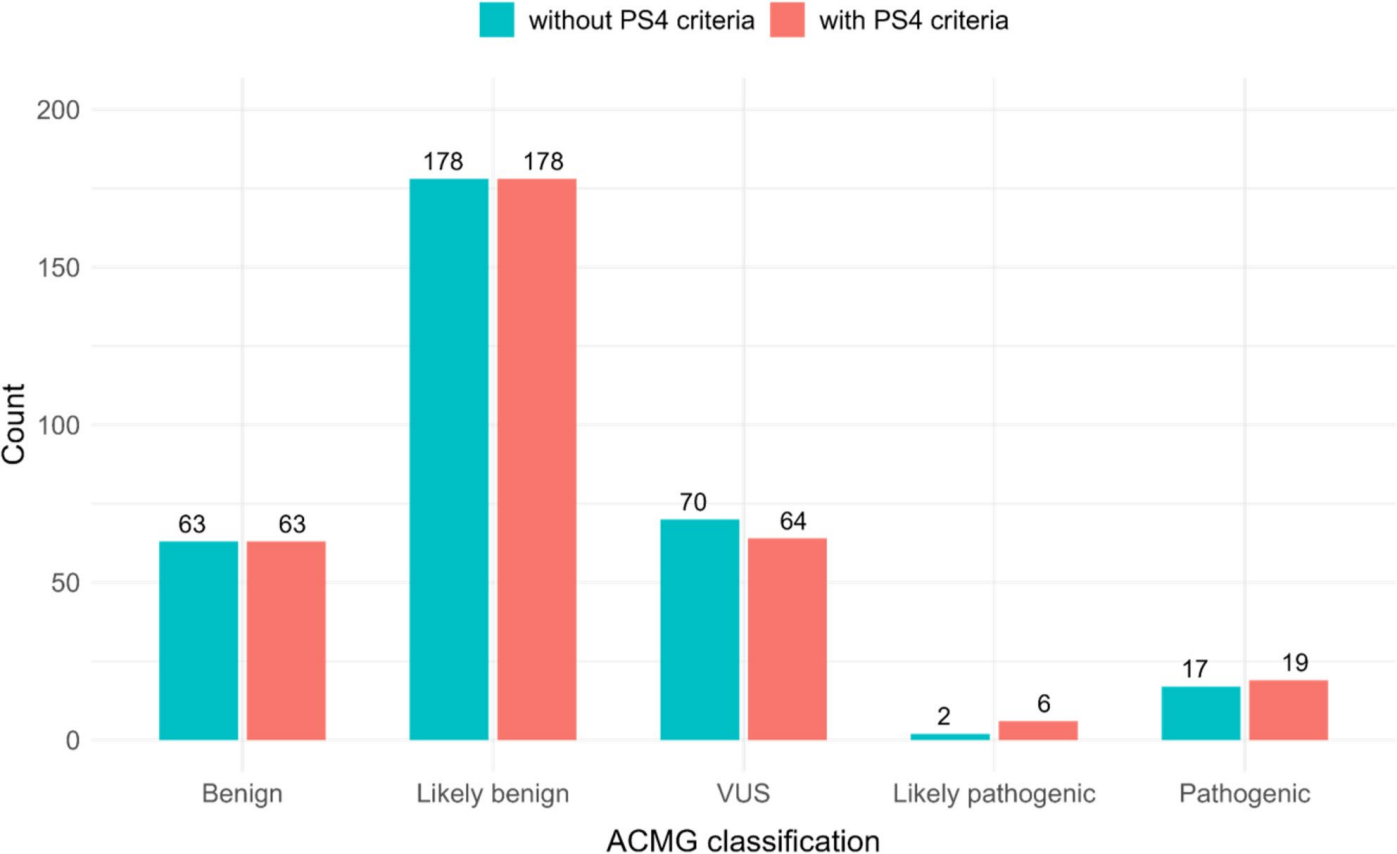
Effect of PS4 evidence on ACMG/AMP classifications. Comparison of variant classifications before (teal) and after (salmon) applying PS4 evidence derived from case–control allele enrichment against Taiwan Biobank whole-genome reference data. Incorporation of PS4 reduced the number of variants of uncertain significance (VUS) and increased Likely pathogenic and Pathogenic designations following manual curation. Exact counts are listed in Supplementary Tables S3 and S4.

Using the predefined PS4 framework, we identified variants in the TIP cohort that were significantly enriched in cases compared with population-matched controls, followed by post-PS4 filtering as described in the Methods. This two-step triage markedly reduced the number of candidate variants while retaining those with plausible molecular and clinical relevance. The impact of PS4 on categorical variant interpretation is summarized in Fig. 1 and detailed in Supplementary Tables S3 and S4. Baseline ACMG/AMP evidence tags and provisional classifications were first generated using GeneBe without PS4, after which variants were manually reviewed to assess whether case–control enrichment, considered together with phenotype and inheritance consistency, supported reclassification.

Incorporation of PS4 led to clinically meaningful upgrades for a subset of IRD-associated variants. Two variants originally classified as Likely pathogenic were upgraded to Pathogenic (*CYP4V2*:c.802_810delinsGC and *CYP4V2*:c.1507G > C). In addition, six variants that were classified as Variant of Uncertain Significance (VUS) in the baseline GeneBe-based workflow were upgraded to Likely pathogenic after incorporation of PS4 evidence, including *CFAP410*:c.340_351dup, *EYS*:c.5644 + 5G > A, *USH2A*:c.13339 A > G, *CNGB1*:c.2921T > G, *REEP6*:c.223G > A, and *USH2A*:c.2802T > G (Table 1; Supplementary Table S3). By contrast, the proportions of Benign and Likely benign classifications remained largely unchanged. Collectively, these findings demonstrate that incorporation of PS4 provides important additional evidence for a subset of variants and improves classification resolution in this panel-sequenced IRD cohort. For variants located on chromosome X, additional sex-stratified PS4 analysis was performed to account for sex-specific ploidy and cohort composition, yielding more biologically appropriate enrichment signals for X-linked variants, including *RS1*(NM_000330):c.531T > G (Table 1; Supplementary Table S4). As a sensitivity analysis, false discovery rate (FDR) adjusted q-values were calculated across all variants subjected to PS4 testing. A substantial subset of PS4-enriched variants remained significant after FDR correction (q < 0.05; Supplementary Table S5), supporting the robustness of these findings under multiple testing control.

**Table 1:** Clinically classified variant enrichment: TIP cohort vs. Taiwan biobank

Gene	Variant	Case AC/AN (TIP cohort)	Control AC/AN (Taiwan Biobank)		OR (95% CI)	ACMG classification^a^	ClinVar^b^
*EYS*	NM_001142800.2:c.7492G > C	13/1604	0/2984		50.6 (3.01-852.33)	P	P/LP
*PROM1*	NM_006017.3:c.139del	10/1604	0/2984		39.3 (2.30-671.24)	P	P/LP
*ABCA4*	NM_000350.3:c.5761G > A	6/1604	0/2984		24.3 (1.37-431.15)	P	P/LP
*RS1*	NM_000330.4:c.531T > G	3/262 (XY) 0/532 (XX)	0/744 (XY) 1/1496 (XX)		20.1 (1.03-390.13) 0.9 (0.04–23.02)	P	P/LP
*ABCA4*	NM_000350.3:c.1804 C > T	15/1604	1/2984		19.4 (3.63-103.77)	P	P/LP
*RDH12*	NM_152443.3:c.505 C > G	12/1604	1/2984		15.6 (2.87–85.01)	P	P/LP
*EYS*	NM_001142800.2:c.6416G > A	62/1604	8/2984		14.2 (6.91–29.13)	P	P/LP
*EYS*	NM_001142800.2:c.8012T > A	9/1604	1/2984		11.8 (2.11–66.34)	P	P/LP
*ABCA4*	NM_000350.3:c.2894 A > G	12/1604	2/2984		9.4 (2.41–36.46)	P	P
*USH2A*	NM_206933.4: c.4576G > A	10/1604	2/2984		7.9 (1.98–31.25)	P	P
*EYS*	NM_001142800.2: c.7228 + 1G > A	18/1604	4/2984		7.7 (2.75–21.68)	P	P/LP
*RCBTB1*	NM_018191.4:c.170del	5/1482	1/2984		7.4 (1.22–45.08)	P	P/LP
*USH2A*	NM_206933.4:c.9570 + 1G > A	9/1604	2/2984		7.1 (1.76–28.65)	P	P
*ABCA4*	NM_000350.3: c.101_106del	5/1604	1/2984		6.8 (1.12–41.64)	P	P
*RLBP1*	NM_000326.5:c.282del	18/1604	6/2984		5.3 (2.18–13.09)	P	P/LP
*CYP4V2*	NM_207352.4:c.802-8_810delinsGC	34/1604	12/2984		5.2 (2.73–10.01)	LP > P	P/LP
*BBS10*	NM_024685.4:c.445dup	6/1604	0/2984		24.3 (1.37-431.15)	P	Conflicting
*POC1B*	NM_172240.3:c.419dup	6/1604	0/2984		24.3 (1.37-431.15)	P	No reported
*CYP4V2*	NM_207352.4:c.1507G > C	6/1604	1/2984		8.1 (1.37–47.79)	LP > P	VUS
*CFAP410*	NM_004928.3:c.340_351dup	7/1090	0/2984		41.3 (2.36-724.07)	VUS > LP	VUS
*EYS*	NM_001142800.2:c.5644 + 5G > A	16/1604	1/2984		20.7 (3.88-110.04)	VUS > LP	P/LP
*USH2A*	NM_206933.4: c.13339 A > G	9/1604	2/2984		7.1 (1.76–28.65)	VUS > LP	Conflicting
*CNGB1*	NM_001297.5:c.2921T > G	8/1604	2/2984		6.4 (1.55–26.05)	VUS > LP	Conflicting
*REEP6*	NM_138393.4:c.223G > A	5/1040	2/2984		6.3 (1.42–28.30)	VUS > LP	Conflicting
*USH2A*	NM_206933.4:c.2802T > G	22/1604	7/2984		5.6 (2.46–12.93)	VUS > LP	Conflicting

AC: allele count, AN: allele number, OR: odds ratio, P: pathogenic, LP: likely pathogenic, VUS: variant of uncertain significance

^a^ACMG classification was assigned using GeneBe following the ACMG/AMP criteria. ^b^ClinVar status is based on the ClinVar database update on 2025-07-21

### ClinVar concordance and cohort-specific refinements

To contextualize Table 1, and recognizing that ClinVar evidence is incorporated into the baseline GeneBe classification through PP5, we additionally compared PS4-enriched variants with current ClinVar entries (accessed July 21, 2025) to assess concordance with publicly available interpretations and to identify variants for which cohort-specific PS4 evidence provides refinement beyond existing submissions. Among these variants, 17 already had ClinVar submissions designating them as Pathogenic or Likely pathogenic, which was consistent with our classifications. Notably, 10 of these 17 variants were located in three genes that are most frequently implicated in Taiwanese IRD cohorts: *EYS*, *USH2A*, and *ABCA4* [11]. In contrast, eight variants were listed in ClinVar as VUS, conflicting, or not reported. Three of these, *BBS10*:c.445dup, *POC1B*:c.419dup, and *CYP4V2*:c.1507G > C, were classified by our analysis pipeline as Likely pathogenic or higher even before PS4 evidence was incorporated, whereas ClinVar assertions were uncertain or absent. Across the TIP cohort, eight patients carried one of these three variants. Two individuals were homozygous for *BBS10*:c.445dup, three carried *CYP4V2*:c.1507G > C (a variant modeled to disrupt an α-helical segment and impair protein function) [14], and three were diagnosed with *POC1B*-related disease. These were the only *POC1B* diagnoses in the cohort, and all three affected individuals harbored the c.419dup variant as one of the disease-associated alleles, indicating that this variant is a recurrent pathogenic allele in this population. Overall, these comparisons indicate that most high-confidence variants align with existing ClinVar designations, whereas a substantial subset benefits from PS4-supported evidence and cohort-specific observations. This additional evidence enables reclassification of previously uncertain or conflicting variants toward clinically actionable interpretation.

### Homozygous cases supporting PS4-driven reclassification

To further clarify the contribution of monoallelic and biallelic carriers to PS4-enriched variants, we summarized the diagnostic status of probands carrying variants listed in Table 1, including monoallelic and biallelic configurations, as shown in Supplementary Fig. S1. Here, we focus on homozygous exemplars that provided strong genotype–phenotype corroboration beyond statistical enrichment. Among the six variants that advanced from VUS to Likely pathogenic after PS4 integration, our cohort included homozygous carriers of two variants, *CNGB1*:c.2921T > G and *CFAP410*:c.340_351dup, which offered direct clinical and molecular support for reclassification. For two additional variants (*USH2A*:c.13339 A > G and *REEP6*:c.223G > A), orthogonal validation and phenotype concordance were confirmed in representative cases and are summarized in Supplementary Fig. S2. For the remaining two variants (*USH2A*:c.2802T > G and *EYS*:c.5644 + 5G > A), molecular and clinical evidence has been established in our previous studies and was therefore not duplicated here [15, 16], but incorporated into manual ACMG/AMP curation.

**Fig. 2:**
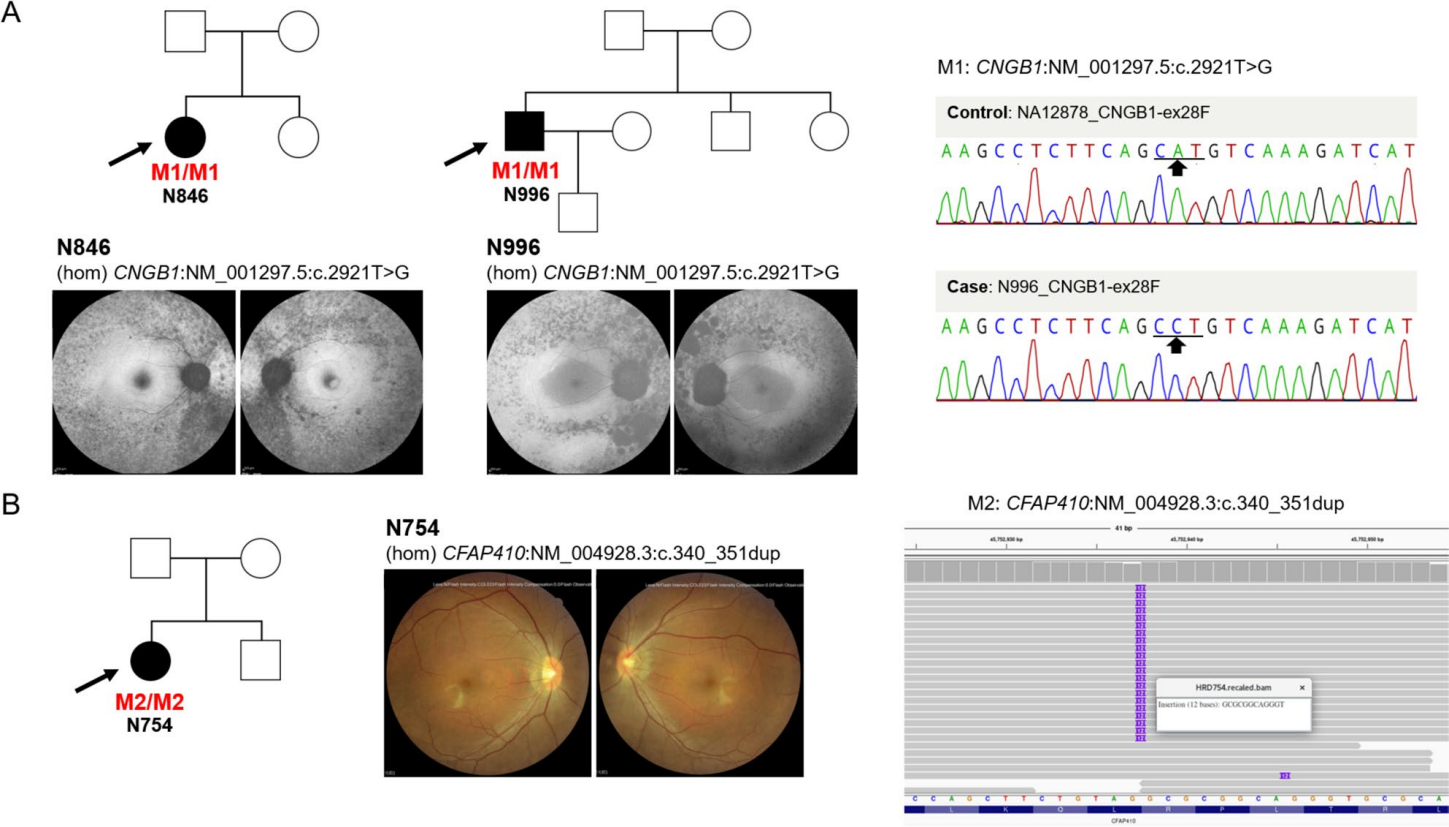
Pedigrees, retinal imaging, and molecular validation for homozygous IRD variants. **A**, *CNGB1*-affected cases with variant NM_001297.5:c.2921T > G. Pedigrees of two unrelated families carrying the homozygous *CNGB1* variant are shown, with probands indicated by arrows. Representative fundus autofluorescence images from the probands display diffuse pigmentary changes with macular involvement, findings consistent with a rod–cone dystrophy pattern. Sanger sequencing of proband N996 confirms *CNGB1*(NM_001297.5):c.2921T > G, with the altered nucleotide indicated; a control chromatogram is provided for comparison. **B**, *CFAP410*-affected case with variant NM_004928.3:c.340_351dup. The pedigree of a family harboring the homozygous *CFAP410* small insertion–duplication is shown. Color fundus photographs from the proband demonstrate bilateral retinal abnormalities. Short-read alignment visualized in the Integrative Genomics Viewer (IGV) validates *CFAP410*(NM_004928.3):c.340_351dup, with supporting reads spanning the duplicated segment

Two unrelated families in our cohort carried the same homozygous *CNGB1*:c.2921T > G variant, with pedigrees and probands shown in Fig. 2A (top). In both probands, fundus autofluorescence imaging demonstrated diffuse pigmentary change with macular involvement (Fig. 2A, middle), and each reported night blindness, which is characteristic of *CNGB1*-associated retinopathy. In proband N996, Sanger sequencing of *CNGB1* exon 28 confirmed the c.2921T > G substitution (Fig. 2A, right), providing independent validation of the next-generation sequencing finding. Additional validation using the Integrative Genomics Viewer (IGV) is provided for the two independent *CNGB1*-diagnosed cases in Supplementary Fig. S3 to further support reproducibility [17]. The homozygous state was consistent with the known recessive inheritance pattern of *CNGB1* retinopathy, and the clinical presentation, characterized by night blindness with compatible imaging, supported phenotype specificity (PP4). Together with case–control enrichment (PS4), these findings justified reclassification from VUS to Likely pathogenic in our analysis.

In a separate family, the proband carried a homozygous *CFAP410*:c.340_351dup variant (Fig. 2B, left) and exhibited early-onset disease with nystagmus. Bilateral color fundus photographs showed retinitis pigmentosa (RP)-like pigmentary change with relative macular preservation (Fig. 2B, middle), and full-field electroretinography demonstrated flat cone responses with generalized photoreceptor dysfunction. Taken together, these findings are consistent with a rod–cone dystrophy (RCD) pattern. Short-read alignment in the IGV revealed a read-supported 12-bp duplication at the annotated locus (Fig. 2B, right), confirming the variant at the sequence level. These clinical and molecular features closely mirror those reported for *CFAP410*-associated retinopathy [18], supporting genotype–phenotype concordance and, together with PS4 enrichment, enabling reclassification from VUS to Likely pathogenic.

These two homozygous exemplars, each consistent with the expected recessive inheritance model and supported by retinal imaging and independent sequencing, demonstrate how PS4-driven enrichment, when integrated with orthogonal molecular validation and clinical context, can shift variants across interpretive thresholds and lead to clinically meaningful classifications in IRD panel sequencing. Importantly, such reclassification from VUS to Likely Pathogenic provides a definitive molecular diagnosis for affected individuals, which is a prerequisite for genetic counseling and for consideration of gene-specific management or clinical trial eligibility in inherited retinal disorders.

## Discussion

This work provides one of the earliest systematic evaluations of the operational application of PS4 for IRD variant interpretation in a population-matched, panel-sequenced cohort of modest size. Although our case number is not large by international standards, an 802-case cohort is substantial and clinically representative within Taiwan. Pairing it with 1,492 ancestry-matched controls enabled robust case–control enrichment. By prespecifying thresholds (odds ratio ≥ 5 with the 95% confidence interval excluding 1), implementing joint genotyping, and applying a light and transparent post-PS4 triage, we demonstrate that a clearly defined, ancestry-matched PS4 workflow can contribute to classification refinement even when sample sizes are constrained. This process facilitates systematic evaluation of uncertain variants toward reportable findings while maintaining specificity. Our experience offers a practical template for centers with limited caseloads: use high-quality local controls, standardize PS4 computation and evidence mapping, and integrate enrichment with phenotype and inheritance checks.

Importantly, these observations underscore that PS4 does not operate in isolation and must be interpreted alongside population frequency criteria and other ACMG/AMP evidence. Among the variants that met PS4 criteria, the largest proportion was ultimately classified as Benign or Likely benign (Fig. 1), including a subset with apparently elevated odds ratios. Upon re-review, most of these high-odds-ratio sites also satisfied BA1 or BS1 population criteria, which appropriately outweighed PS4. Notably, BA1 and BS1 are typically evaluated against the highest reliable population frequency, such as gnomAD popmax, and against a disease-specific maximum credible allele frequency, which is often well below 1% for Mendelian disorders [19]. As a result, a variant may appear rare in East Asians and pass our post-PS4 East Asian frequency filter but still be common in another ancestry or exceed the disorder-specific ceiling. This pattern can yield a Benign or Likely benign classification despite an inflated odds ratio in our cohort. Cohort scale can also influence this effect. With 802 cases and 1,492 controls, stochastic variation and subtle population stratification can exaggerate odds ratios for otherwise tolerated alleles. Supporting this interpretation, a recent Japanese IRD guideline recommends that ancestry-matched cohorts include more than 20,000 individuals to achieve robust PS4 assessment. Larger sample sizes help stabilize effect estimates and reduce spurious enrichment caused by small numbers and substructure [20]. Consequently, while our design is useful for hypothesis generation and triage, larger ancestry-matched resources combined with popmax-aware and condition-specific frequency thresholds will be essential to reduce the proportion of high-odds-ratio variants ultimately classified as Benign or Likely benign. Such expansion will also support the use of graded PS4 strengths with greater confidence.

Evolving guidance in the field underscores that there is no single universal strategy for operationalizing PS4 across diseases. When well-powered case–control data are unavailable, several ClinGen Variant Curation Expert Panels (VCEPs) assign PS4 using affected-proband counts with gene- or phenotype-specific thresholds, as exemplified by approaches adopted in RASopathy [21]. In IRD, disease- and gene-specific adaptations of PS4 have likewise been proposed, particularly for X-linked conditions such as *RPGR*-associated disease, where conventional case–control designs may be impractical [22]. In addition to proband-count-based approaches, recent large-scale studies have demonstrated that PS4 can be calibrated directly from quantitative enrichment data derived from ancestry-matched case-control cohorts. In the context of hereditary hearing loss, odds-ratio- and likelihood-ratio-based thresholds have been derived from large numbers of cases and controls, enabling the assignment of graded PS4 strengths (supporting, moderate, and strong) according to observed variant frequencies and suggesting that such quantitative frameworks may be applicable to other monogenic disorders [23]. Importantly, comparative analyses across multiple disease domains have further shown that the odds-ratio thresholds required to reach a given PS4 tier differ by phenotype, indicating that PS4 calibration should be tailored to specific disease contexts rather than applied uniformly across conditions [24]. Collectively, these studies highlight that PS4 is inherently context-dependent, influenced by gene, phenotype, genetic architecture, and the availability of appropriately matched control data. This perspective provides a strong rationale for our ancestry-matched, explicitly defined PS4 framework in IRD, which leverages population-specific controls to generate interpretable and reproducible enrichment evidence within a clinically relevant setting.

This study has some limitations. First, although we used a standardized DRAGEN/GRCh38 pipeline with joint genotyping, differences in panel versions and sequencing batches may affect variant callability and result in non-uniform allele numbers (AN). Such inconsistencies can introduce minor biases in case-control counts and odds-ratio estimates. This impact is likely greater for intronic or intergenic variants due to more variable capture efficiency and coverage; therefore, detailed analyses of intronic or intergenic variants were not included in this study. Future work using whole-genome sequencing may enable more robust assessment of these regions. Second, statistical power remains a challenge in rare variant analysis. As shown in our sensitivity analyses, stringent multiple-testing correction (such as FDR) may reduce the apparent significance of ultra-rare variants, even when they show strong effect sizes. To address this, variants that did not pass FDR thresholds were interpreted only when supported by independent evidence, including segregation, or consistent clinical phenotypes. Larger ancestry-matched cohorts will be required in future studies to enable more definitive statistical evaluation of such rare variants without reliance on additional supporting data. Third, we applied a fixed PS4 criterion (odds ratio ≥ 5 with the 95% confidence interval excluding 1) following the 2015 ACMG/AMP guidelines. Emerging disease-specific frameworks recommend phenotype-dependent OR thresholds and graded PS4 strengths, meaning some clinically relevant variants might be under- or misclassified. Adopting these refined approaches in future analyses could improve classification performance.

In conclusion, this study demonstrates that a practical, ancestry-matched PS4 workflow can enhance variant interpretation in IRD, particularly in mid-sized cohorts. By integrating predefined enrichment criteria with phenotype and inheritance concordance, this workflow reduces the burden of variants of uncertain significance and improves the clinical utility of panel-based sequencing. The framework is readily adoptable by other IRD programs and extensible to additional monogenic disorders with well-characterized case cohorts and appropriate population reference data. Future efforts should focus on expanding ancestry-matched reference datasets, harmonizing technical metrics such as per-variant allele numbers, and implementing disease-aware, graded PS4 thresholds to further improve diagnostic yield and clinical impact.

## Supplementary Information

Below is the link to the electronic supplementary material.

Supplementary Material 1.

Supplementary Material 2.

## Data Availability

Variant summary data appear in Supplementary Tables S3 and S4, and raw VCFs are available from the corresponding author upon request.

## References

[1] Sahel JA, Marazova K, Audo I. Clinical characteristics and current therapies for inherited retinal degenerations. Cold Spring Harb Perspect Med. 2015;5:a017111. 10.1101/cshperspect.a017111 PMC4315917 25324231

[2] RetNet. Disease Table [Internet]. [cited 2023 Mar 29];Available from: https://web.sph.uth.edu/RetNet/disease.htm

[3] Liu X, Xiao J, Huang H, Guan L, Zhao K, Xu Q, et al. Molecular genetic testing in clinical diagnostic assessments that demonstrate correlations in patients with autosomal recessive inherited retinal dystrophy. JAMA Ophthalmol. 2015;133:427–36. 25611614 10.1001/jamaophthalmol.2014.5831

[4] Bertelsen M, Jensen H, Bregnhøj JF, Rosenberg T. Prevalence of generalized retinal dystrophy in Denmark. Ophthalmic Epidemiol. 2014;21:217–23. 24963760 10.3109/09286586.2014.929710

[5] Kim YJ, Kim YN, Yoon YH, Seo EJ, Seo GH, Keum C, et al. Diverse genetic landscape of suspected retinitis pigmentosa in a large Korean cohort. Genes. 2021;12:675. 33946315 10.3390/genes12050675 PMC8146864

[6] Suga A, Yoshitake K, Minematsu N, Tsunoda K, Fujinami K, Miyake Y, et al. Genetic characterization of 1210 Japanese pedigrees with inherited retinal diseases by whole-exome sequencing. Hum Mutat. 2022;43:2251–64. 36284460 10.1002/humu.24492

[7] Cho SY, Hong EJ, Nam JM, Han B, Chu C, Park O. Opening of the National biobank of Korea as the infrastructure of future biomedical science in Korea. Osong Public Health Res Perspect. 2012;3:177–84. 24159511 10.1016/j.phrp.2012.07.004 PMC3738708

[8] Nagai A, Hirata M, Kamatani Y, Muto K, Matsuda K, Kiyohara Y, et al. Overview of the biobank Japan project: study design and profile. J Epidemiol. 2017;27:S2–8. 28189464 10.1016/j.je.2016.12.005 PMC5350590

[9] Laugesen K, Mengel-From J, Christensen K, Olsen J, Hougaard DM, Boding L, et al. A review of major Danish biobanks: advantages and possibilities of health research in Denmark. Clin Epidemiol. 2023;15:213–39. 36852012 10.2147/CLEP.S392416 PMC9960719

[10] Richards S, Aziz N, Bale S, Bick D, Das S, Gastier-Foster J, et al. Standards and guidelines for the interpretation of sequence variants: A joint consensus recommendation of the American college of medical genetics and genomics and the association for molecular pathology. Genet Med. 2015;17:405–24. 25741868 10.1038/gim.2015.30 PMC4544753

[11] Chen TC, Huang DS, Lin CW, Yang CH, Yang CM, Wang VY, et al. Genetic characteristics and epidemiology of inherited retinal degeneration in Taiwan. Npj Genom Med. 2021;6:1–8. 33608557 10.1038/s41525-021-00180-1 PMC7896090

[12] Hsu JS, Wu DC, Shih SH, Liu JF, Tsai YC, Lee TL, et al. Complete genomic profiles of 1496 Taiwanese reveal curated medical insights. J Adv Res. 2024;66:197–207. 38159844 10.1016/j.jare.2023.12.018 PMC11675050

[13] Wang K, Li M, Hakonarson H. ANNOVAR: functional annotation of genetic variants from high-throughput sequencing data. Nucleic Acids Res. 2010;38:e164. 20601685 10.1093/nar/gkq603 PMC2938201

[14] Chan LW, Sung YC, Wu DC, Chen CY, Yang CH, Yang CM, et al. PREDICTED PROTEIN STRUCTURE VARIATIONS INDICATE THE CLINICAL PRESENTATION OF CYP4V2-RELATED BIETTI CRYSTALLINE DYSTROPHY. RETINA. 2022;42:797. 34923510 10.1097/IAE.0000000000003381

[15] Lin YW, Huang YS, Lin CY, Lin CW, Wu CC, Yang CH, et al. High prevalence of exon-13 variants in USH2A-related retinal dystrophies in Taiwanese population. Orphanet J Rare Dis. 2024;19:238. 38879497 10.1186/s13023-024-03238-2 PMC11179209

[16] Huang YS, Lu WT, Chiu IH, Chien PM, Chen YA, Lin CY, et al. Hidden splicing variants in inherited retinal degeneration: discovery and functional insight. Invest Ophthalmol Vis Sci. 2025;66:12. 41060150 10.1167/iovs.66.13.12 PMC12517372

[17] Thorvaldsdóttir H, Robinson JT, Mesirov JP. Integrative genomics viewer (IGV): high-performance genomics data visualization and exploration. Brief Bioinform. 2013;14:178–92. 22517427 10.1093/bib/bbs017 PMC3603213

[18] Sangermano R, Gupta P, Price C, Han J, Navarro J, Condroyer C, et al. Coding and non-coding variants in the ciliopathy gene CFAP410 cause early-onset non-syndromic retinal degeneration. Npj Genom Med. 2024;9:58. 39516462 10.1038/s41525-024-00439-3 PMC11549414

[19] Whiffin N, Minikel E, Walsh R, O’Donnell-Luria AH, Karczewski K, Ing AY, et al. Using high-resolution variant frequencies to empower clinical genome interpretation. Genet Med. 2017;19:1151–8. 28518168 10.1038/gim.2017.26 PMC5563454

[20] Fujinami K, Nishiguchi KM, Oishi A, Akiyama M, Ikeda Y, Hotta Y, et al. Specification of variant interpretation guidelines for inherited retinal dystrophy in Japan. Jpn J Ophthalmol. 2024;68:389–99. 39078460 10.1007/s10384-024-01063-5

[21] Wilcox EH, Webb RF, Tshering KC, Hughes MY, Cavé H, DiStefano MT, et al. Updated ACMG/AMP specifications for variant interpretation and gene curations from the ClinGen rasopathy expert panels. Genet Med Open. 2025;3:103430. 40496714 10.1016/j.gimo.2025.103430 PMC12151217

[22] Worley KC, Mero M, Hankey W, Haer-Wigman L, Hussain HMJ, Lee K, et al. ClinGen X-linked inherited retinal disease variant curation expert panel (XLIRD VCEP): RPGR curation Progress, and initial modified ACMG criteria for RS1 and CHM. Invest Ophthalmol Vis Sci. 2024;65:453.

[23] Liu S, Zhong M, Huang Y, Zhang Q, Chen T, Xu X, et al. Quantitative thresholds for variant enrichment in 13,845 cases: improving pathogenicity classification in genetic hearing loss. Genome Med. 2023;15:116. 38111038 10.1186/s13073-023-01271-7 PMC10726519

[24] Bhat V, Yu T, Brown L, Pejaver V, Lebo M, Harrison S, et al. Extracting and calibrating evidence of variant pathogenicity from population biobank data. Am J Hum Genet. 2025;112:1805–17. 40639380 10.1016/j.ajhg.2025.06.012 PMC12401458

