# Supplementary material for: From enrichment to interpretation: PS4-driven reclassification in Taiwanese inherited retinal degeneration
Source: Hum Genomics. 2026 Feb 15;20:56. doi: 10.1186/s40246-026-00923-0 (PMC13011756; doi:10.1186/s40246-026-00923-0)
Supplement: Supplementary file 1 — Supplementary Material 1. [file 40246_2026_923_MOESM1_ESM.docx]

**Supplementary Figures**


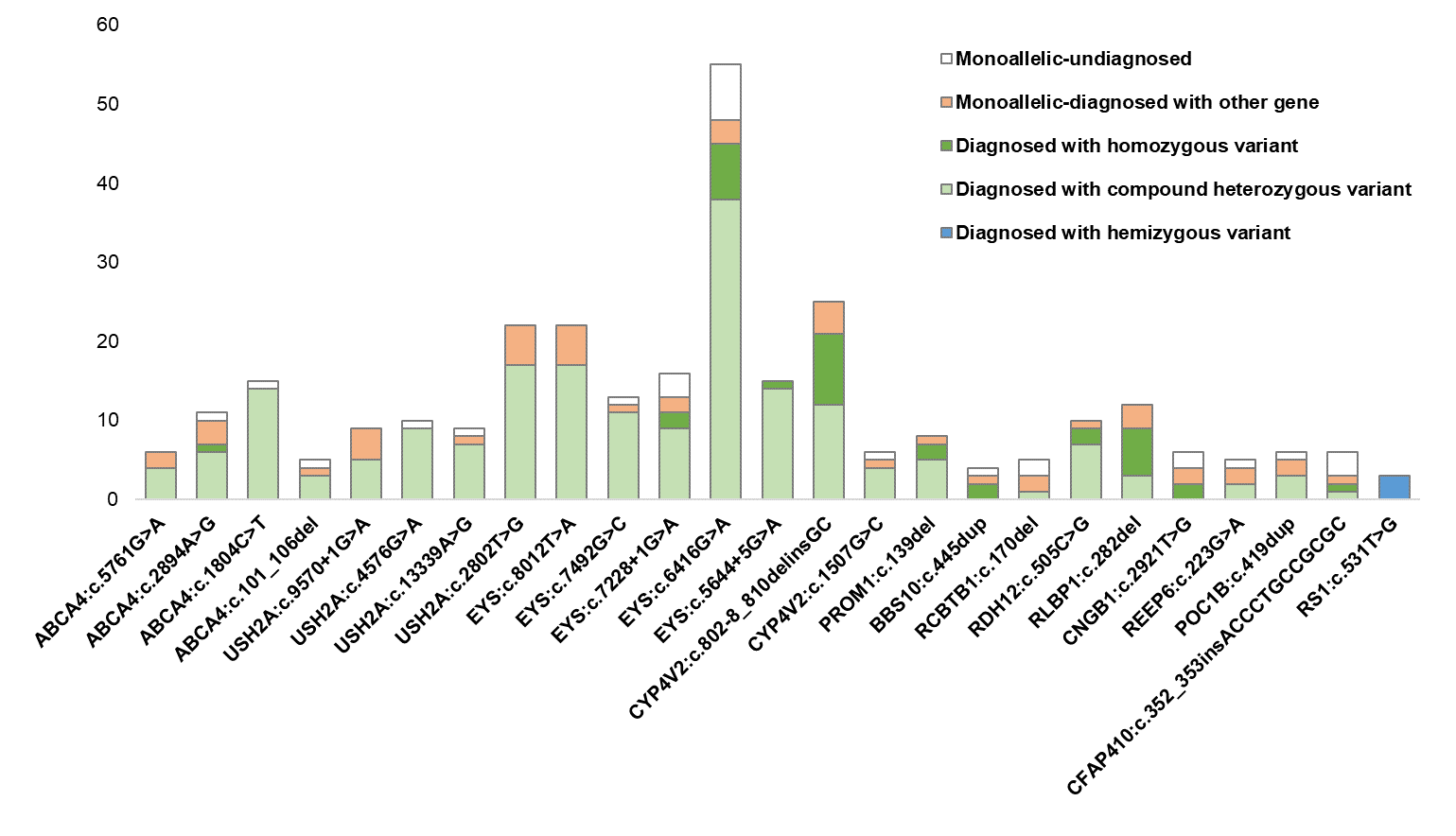


**Supplementary Fig. S1 Distribution of diagnostic status among carriers of PS4-enriched variants in the TIP IRD cohort.**
Stacked bar chart showing the number of individuals carrying each PS4-enriched variant in the TIP IRD cohort, stratified by diagnostic status. Variants are shown on the x-axis, and the y-axis indicates the number of individuals. Bars are color-coded according to clinical interpretation: monoallelic undiagnosed, monoallelic diagnosed with another gene, diagnosed with homozygous variant, diagnosed with compound heterozygous variant, and diagnosed with hemizygous variant. Each bar represents the total number of carriers for a given variant, with stacked segments indicating the distribution of zygosity and diagnostic context.


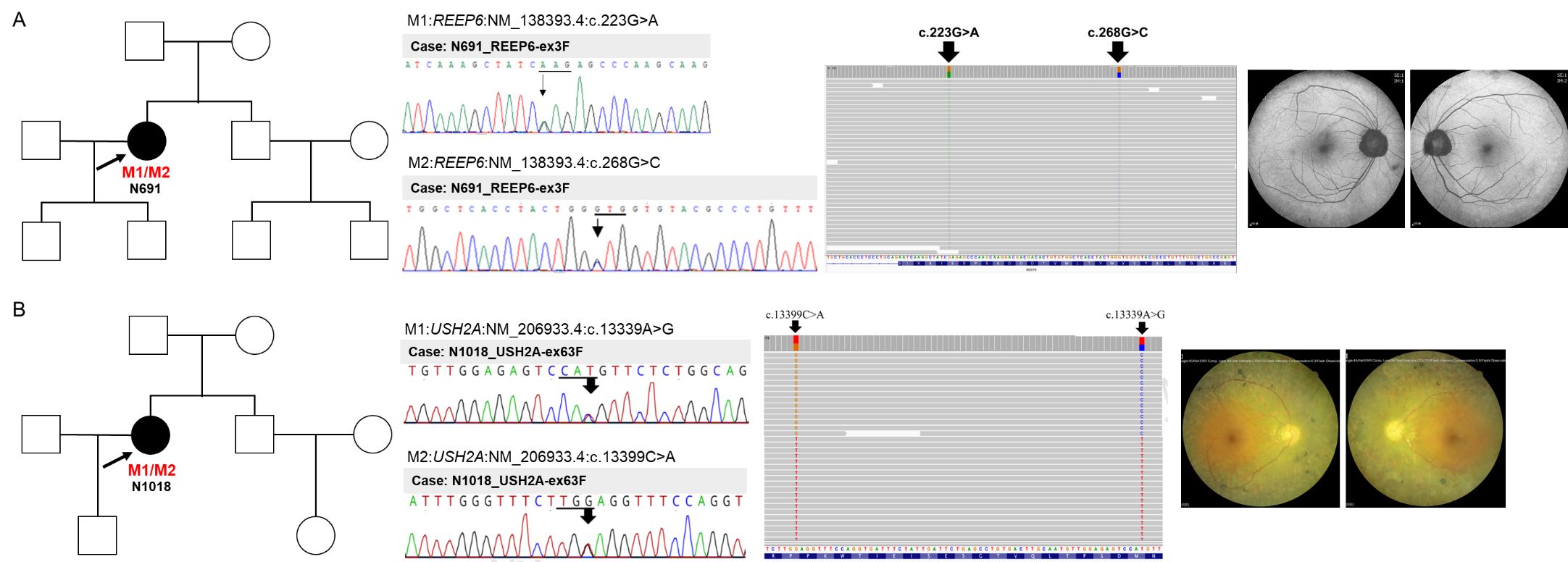


**Supplementary Fig. S2. Orthogonal validation, phasing, and phenotype concordance for representative VUS-to-Likely Pathogenic variants.**

(A) Case N691, carrying compound heterozygous pathogenic variants in *REEP6* (NM_138393.4), including c.223G>A and c.268G>C. Phasing analysis confirmed that the two variants are located on different alleles. Sanger sequencing chromatograms and Integrative Genomics Viewer (IGV) screenshots validate the presence of both variants. Clinically, the patient reported nyctalopia and progressive bilateral blurred vision, and ophthalmic examinations revealed peripheral bone-spicule pigmentation, retinal pigment epithelium changes, and reduced electroretinogram responses, consistent with *REEP6*-related inherited retinal disease. (B) Case N1018, harboring compound heterozygous pathogenic variants in *USH2A* (NM_206933.4), including c.13339A>G and c.20693G>A. Phasing confirmed that the variants are in trans. Sanger sequencing and IGV analysis support the identified variants. Clinically, the patient reported disease onset in the third decade of life, and ophthalmic examinations revealed peripheral retinal pigmentation with relative preservation of central retinal structure and visual function, consistent with *USH2A*-related inherited retinal disease.


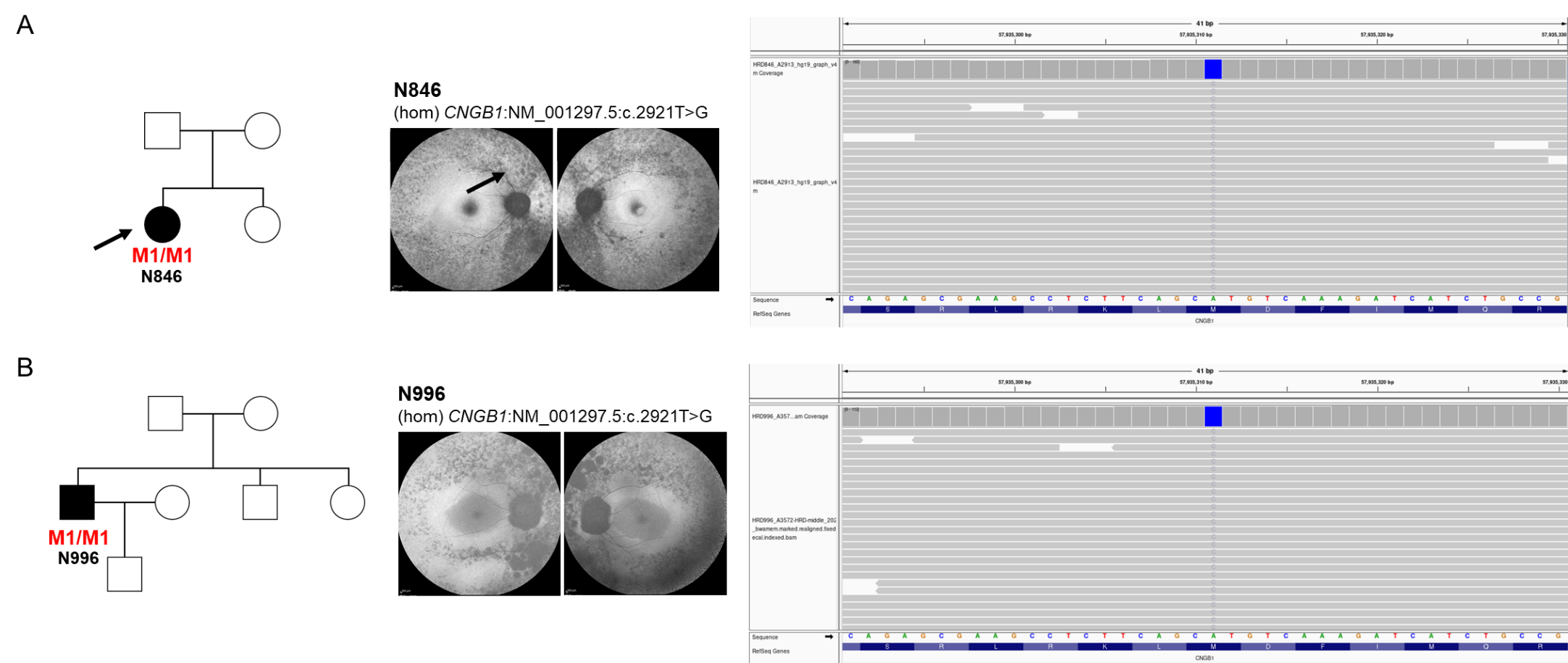


**Supplementary Figure S3. Orthogonal validation and clinical concordance for homozygous *CNGB1*:c.2921T>G cases.**

Two unrelated probands diagnosed with *CNGB1*-associated retinopathy are shown. (A) Representative case N846 and (B) representative case N996. Pedigrees indicate homozygous inheritance of the *CNGB1*:c.2921T>G variant in both families. Fundus autofluorescence images demonstrate diffuse retinal degeneration with macular involvement, consistent with the known phenotype of *CNGB1*-related disease. Integrative Genomics Viewer (IGV) snapshots confirm homozygous variant calls with adequate read depth and balanced allele support at the *CNGB1* locus.
